# Feasibility of early spontaneous breathing in acute respiratory distress syndrome

**DOI:** 10.1186/cc10713

**Published:** 2012-03-20

**Authors:** S Mortaza, A Mercat

**Affiliations:** 1 CHU Angers, France

## Introduction

Airway pressure release ventilation (APRV) is as a pressure preset mode that allows unrestricted spontaneous breathing throughout the entire ventilator cycle. In ARDS, this mode may decrease the need for sedation, increase alveolar recruitment and improve hemodynamic tolerance. The aim of this study is to assess the feasibility of a protocol combining APRV settings and sedation adaptation in order to obtain levels of spontaneous breathing between 10 and 50% of total minute ventilation.

## Methods

We designed a monocentric study including 10 patients with early ARDS. We used a Dräger Evita XL ventilator. We initially used the volume-assist control (VAC) mode to set the PEEP, tidal volume and respiratory rate according to the increased recruitment strategy of the ExPress trial [1]. These settings were used to adjust the ventilator's parameters in APRV mode: low pressure at the same level as the PEEP applied in VAC, high pressure (<32 cmH_2_O) set to reach a tidal volume of 6 ml/kg of predicted body weight (PBW), T high between 0.8 and 1 second, T low set to reach the same respiratory rate as in VAC. Patients were initially paralyzed. Then sedation was adapted to keep RASS ≥-4 and a level of spontaneous breathing between 10 and 50% of total minute ventilation. A computer was continuously connected to the ventilator for 5 days in order to record respiratory variables.

## Results

**Figure 1:**
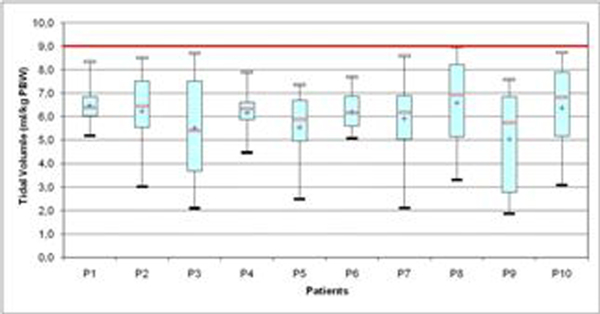
**Tidal volume distribution (ml/kg PBW) for 5 days for each patient**.

At inclusion, baseline characteristics of the patients were the following: five men and five women, 59 years old on average (25 to 85), SAPS II score of 42 (20 to 71), PO_2_/FiO_2 _ratio of 107 (74 to 175) and respiratory system compliance (Cst,rs) of 25 ml/cmH_2_O (18 to 36). We did not observe any pneumothorax. Eight of patients had RASS ≥-4 during the 5 days of enrollment. From day 2, the level of spontaneous breathing ranged between 10 and 50% of total minute ventilation in eight patients. The tidal volume (spontaneous and mechanical) measured for 5 days for each patient was mainly distributed around 6 ml/kg PBW (Figure 1). The mean PO_2_/FiO_2 _ratio increased from 107 ± 29 at enrollment to 173 ± 91 at day 5 (*P *< 0.05).

## Conclusion

APRV settings and the protocol for sedation used in this trial allowed to reach sufficient spontaneous breathing for the majority of the patients without any major complication and with a tidal volume below 8 ml/kg PBW. For a few patients, spontaneous breathing seems to be hard to obtain, especially within the first 2 days.
